# Autoimmune Progesterone Dermatitis: A Case Report

**DOI:** 10.1055/s-0039-1678589

**Published:** 2019-04-02

**Authors:** Özhan Özdemir, Gözde Girgin Yahşi, Cemal Reşat Atalay

**Affiliations:** 1 Department of Obstetrics and Gynecology, Ankara Numune Education and Research Hospital, Ankara, Turkey

**Keywords:** autoimmune, dermatitis, progesterone

## Abstract

**Introduction** Autoimmune progesterone dermatitis (APD) is a rare autoimmune dermatosis characterized by recurrent cutaneous and mucosal lesions during the luteal phase of the menstrual cycle that disappear some days after the menses.

**Case Report** A 34-year-old primipara woman with no significant past medical history and no prior exogenous hormone use, who presented with cyclic skin eruptions starting 1 year after the delivery. The lesions occurred ∼ 6 days before the menses and disappeared in between 1 and 2 days after the menstruation ceased. The patient was diagnosed after a positive response to an intradermal test with progesterone and was successfully treated with combined oral contraceptives. The skin eruptions have not returned since the initiation of this therapy.

**Conclusion** Dermatologists, gynecologists, and obstetricians should be aware of this rare entity. Furthermore, if this condition is suspected, a thorough history taking on the menstrual cycle and results of the intradermal progesterone test are mandatory.

## Introduction

Autoimmune progesterone dermatitis (APD) is a rare autoimmune dermatosis characterized by recurrent cutaneous and mucosal lesions during the luteal phase of the menstrual cycle. These lesions usually disappear in between 1 and 2 days after the menstruation ceases. The pathogenesis of this disorder is unclear. This condition is usually considered as an immune reaction to increased levels of endogenous progesterone during the luteal phase of the menstrual cycle.^1^ The prevalence of APD is unknown, and there are ∼ 90 APD cases in the English literature. In the present article, we report a case of APD and review the current literature regarding its clinical features, pathogenesis, diagnosis, and treatment options.

## Case Report

A 34-year-old primipara woman was admitted to the gynecology clinic for an annual control. She had no significant medical history and no prior exogenous hormone use. The patient had a gynecologic history of menarche at the age of 11 and reported 28-day menstrual cycles. The obstetrical history of the patient consisted of a pregnancy resulting in term cesarean delivery. She presented with cyclic eczematous eruptions starting 1 year after the delivery. The lesions occurred ∼ 6 days before the menses and disappeared in between 1 and 2 days after the menstruation ceased. She reported no episodes of skin lesions during the pregnancy or during the breast-feeding period. The physical examination revealed several erythematous macules and patches on the hand, forearms, and elbows. The patient was evaluated by dermatologists. Allergy testing was performed and the results were negative. The patient reported that she had been submitted to a treatment with antihistamines 2 years previously, but this treatment had not improved her symptoms. The pelvic examination and ultrasound assessment were essentially normal. The routine laboratory investigations, the results of the biochemistry, complete blood count, and the hormone profiling revealed no abnormal findings.

**Fig. 1:**
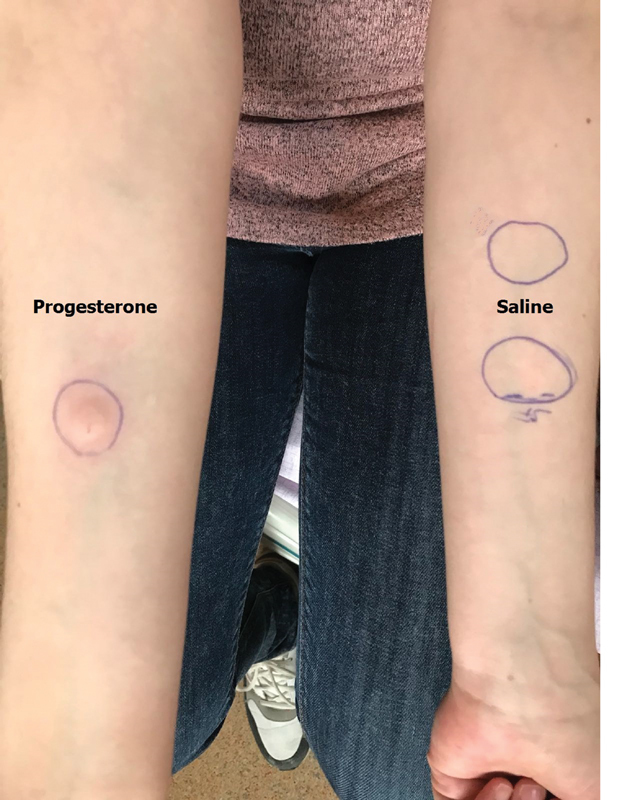
Intradermal testing with progesterone was positive after 15 minutes.

**Fig. 2:**
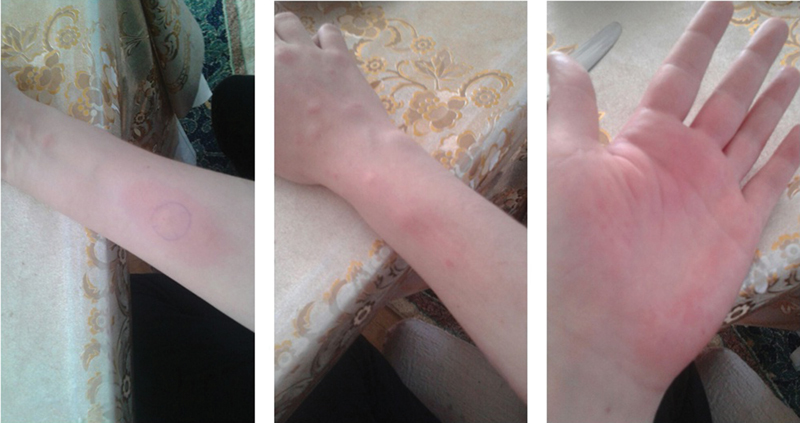
Erythema on forearm and hand 24 hours after progesterone challenge.

We performed an intradermal test using 50 mg/mL progesterone (Progestan, Koçak Farma, Istanbul, Turkey) as a positive control, and normal saline was used as a negative control. Following the first 15 minutes, a 3 cm wide induration appeared on the right arm of the patient, in which the progesterone was injected (Fig. 1). After 24 hours, a widespread edema, itchy dermatitis, and eruptions were observed on the arm of the patient in which the progesterone was injected (Fig. 2). The test results were evaluated to be positive for progesterone dermatitis. The results revealed a negative immediate response and a positive late response on the left arm of the patient, in which normal saline was injected.

Following the diagnosis, the patient received a combined oral contraceptives therapy with drospirenone (3 mg) and ethinyl estradiol (0.03 mg). Five months after the cessation of the therapy, she remained with no skin lesions.

## Discussion

Autoimmune progesterone dermatitis is a rare condition characterized by recurrent skin eruptions during the luteal phase of the menstrual cycle. The first documented case of cyclic urticaria associated with menses was described by Géber in 1921.^2^ The prevalence of APD is unclear, but ∼ 90 cases have been reported in the English literature.

The pathogenicity of APD is unclear, but it is considered to be caused by an immune reaction to endogenous progesterone during the menstrual cycle. This dermatosis, which is exclusively observed in women in child-bearing age, disappears completely with the menopause, an observation that highlights the importance of hormonal triggers. It has been suggested that previous progesterone exposure (i.e., oral contraceptives, intrauterine devices containing progesterone, menarche, pregnancy) results in the stimulation of a hypersensitivity reaction to the endogenous hormones and subsequently leads to overt disease in predisposed patients. Cross-sensitivity to different steroid groups has been suggested as the mechanism in patients with no prior exposure. A cutaneous patch study with hydrocortisone, 11-deoxycortisol, and 17-α-OH-progesterone, the precursor of progesterone, suggested a possible relationship between the steroid groups.^3^

The clinical presentation of APD is highly polymorphous, with erythematous, macular, papular and/or vesicular rashes, palmoplantar dyshidrosis, urticaria, and erythema multiforme-like lesions. Stomatitis and mucosal lesions are less common. The trunk and limbs are the most characteristically affected sites, but there have been descriptions of cases involving the face, the oral mucosa and lips or the genitals. The lesions typically and constantly relapse during the luteal phase of the menstrual cycle. The symptoms appear between 3 and 10 days prior to the menses and remit shortly after the menstruation.^4^

Due to its rarity, the diagnosis for APD should be excluded. There are no definitive diagnostic laboratory tests or specific histopathological findings. This wide spectrum of clinical presentations often delays the diagnosis.^1^ The diagnostic criteria for autoimmune progesterone dermatitis proposed by Warin^5^ include a) skin lesions associated with the menstrual cycle (premenstrual flare); b) a positive response to the progesterone intradermal test or reproducibility of the rash with the intramuscular test; and c) symptomatic improvement after inhibiting progesterone secretion by suppressing ovulation.^5^ A suspicion of APD should be confirmed using an intradermal skin test with progesterone during the follicular phase of the menstrual cycle. Sometimes, an immediate urticarial type of reaction is observed after between 20 and 30 minutes, but most commonly a delayed hypersensitivity reaction appears after between 48 and 72 hours. A drug challenge test with intramuscular progesterone usually leads to the recurrence of the cutaneous eruption after between 6 and 8 hours.^6^

Prior case reports discuss various treatment modalities (i.e., antihistamines, corticosteroids, combined oral contraceptives, tamoxifen, gonadotropin-releasing hormone [GnRH] agonists, and danazol) to attain disease control.^7^ The treatment for ADP relies on oral antihistamines combined with topical and systemic corticosteroids for symptomatic relief. Autoimmune progesterone dermatitis can be treated or controlled mainly by suppressing ovulation and the production of endogenous progesterone during the luteal phase of the cycle. The initial therapy is combined oral contraceptives. Further, GnRH agonists, danazol, and tamoxifen can be used, including in refractory cases. Bilateral oophorectomy can be an option.^8^

## Conclusion

In conclusion, APD is a rare autoimmune response to endogenous or exogenous progesterone. It may present in different morphological forms, resulting in a delayed diagnosis and misdiagnosis. Premenstrual flare, positive intradermal skin test with progesterone, and prevention of lesions with inhibition of ovulation are the main criteria for the diagnosis of APD. Dermatologists, gynecologists, and obstetricians should be aware of this rare entity. Furthermore, if this condition is suspected, a thorough history taking on the menstrual cycle and results of the intradermal progesterone test are mandatory.
